# The Alien Flora of Australia (AFA), a unified Australian national dataset on plant invasion

**DOI:** 10.1038/s41597-023-02746-3

**Published:** 2023-11-27

**Authors:** I. Martín-Forés, G. R. Guerin, D. Lewis, R. V. Gallagher, M. Vilà, J. A. Catford, A. Pauchard, B. Sparrow

**Affiliations:** 1 School of Biological Sciences, The University of Adelaide, Adelaide, South Australia 5005 Australia; 2 Terrestrial Ecosystem Research Network (TERN), University of Adelaide, Adelaide, South Australia 5005 Australia; 3 Hawkesbury Institute for the Environment, Western Sydney University, Richmond, 2753 New South Wales Australia; 4 Doñana Biological Station – Spanish National Research Council (EBD-CSIC), 41092 Sevilla, Spain; 5 Department of Plant Biology and Ecology, University of Sevilla, 41012 Sevilla, Spain; 6 Department of Geography, King’s College London, London, WC2B 4BG UK; 7 School of Agriculture, Food & Ecosystem Sciences, University of Melbourne, Melbourne, Vic 3010 Australia; 8 Faculty of Forestry Sciences, University of Concepcion, Concepcion, Chile; 9 Institute of Ecology and Biodiversity (IEB), Concepción, Chile

**Keywords:** Invasive species, Conservation biology

## Abstract

Biological invasions are a major threat to Australia. Information on alien flora in Australia is collated independently by different jurisdictions, which has led to inconsistencies at the national level, hampering efficient management. To harmonise different information sources, we present the Alien Flora of Australia (AFA), a nationally unified dataset. To create the AFA, we developed an R script that compares existing data sources (the Australian Plant Census and state and territory censuses), identifies mismatches among them and integrates the information into unified invasion statuses at the national scale. The AFA follows the taxonomy and nomenclature adopted for the Australian Plant Census, introduction status and impact of plants known to occur in Australia. The up-to-date information presented in this dataset can aid early warning of alien species invasions, facilitate decision-making at different levels, and biosecurity at national scale. The associated script is ready to be implemented into new versions of the AFA with updated releases of any of the data sources, streamlining future efforts to track of alien flora across Australia.

## Background & summary

The impact of biological invasions poses one of the greatest threats to nature^1^, with rapidly emerging shifts in species ranges and subsequent biodiversity loss impacting ecosystem services^2^, economies^3^ and human well-being^4^. Of special concern are alien plant invasions, because of the great number of species (~14,000) introduced outside their native range compared to other taxa^5^ with no sign of saturation^6^.

Alien plants can be classified based on their position along the introduction–naturalisation–invasion status continuum^7,8^. According to the unified invasion status framework proposed by Blackburn *et al*. (2011), species introduced in a new region are classified differently depending on the barriers they have overcome, regardless of their impact^8^. As such, casual aliens are those that have been transported beyond the limits of their native range. Only a fraction of casual aliens become naturalised to form self-sustaining populations. Finally, only a fraction of those naturalised become invasive, overcoming local dispersal barriers and spreading in the new region^8^.

High quality, easy-to-access, standardised and unified data sources are essential for assessing and monitoring the status of biological invasions, making future predictions and for prioritizing management actions. While there are many freely accessible online databases, their ultimate value depends on the feasibility of integrating information provided at different spatial scales, following different criteria, or using different nomenclature^9^.

Recently, there have been many initiatives to create and make available large-scale datasets of alien flora. Such is the case, for example, of the Global Naturalized Alien Flora (GloNAF)^10^ at the global scale, or the Inventory of alien invasive species in Europe (DAISIE)^11^.

Australia has the second highest number of endemic plant species in the world^12^ and is one of the world’s biodiversity hotspots and megadiverse countries, which are known to be the most vulnerable to biological invasions^13^. To prevent biological invasions, $13.6 billion are currently invested every year in environmental biosecurity in Australia^14^. Australia’s plant censuses, including information on whether a species is native or introduced, have been developed independently by state and territory government environment departments, which has resulted in inconsistencies at the national level. The only national sources about alien flora that exist in Australia are outdated or apply unclear criteria for species’ inclusion and status^15–17^ (http://wwf.org.au/publications/ListInvasivePlants/).

To overcome mismatches caused by jurisdictional boundaries and enable efficient management, standardised data including invasion status at the national scale are needed.

The most accessible and comprehensive plant census at the national scale is the Australian Plant Census (APC; https://biodiversity.org.au/nsl/services/export/index; accessed February 2022), a nationally accepted taxonomy for vascular plants (angiosperms, ferns and gymnosperms). It is one of the taxonomic resources of the Australian National Species List (auNSL; https://biodiversity.org.au/nsl/) and provides authoritative data for names and published taxon concepts for native and naturalised taxa in Australia. Despite being federally managed and endorsed by the Council of Heads of Australasian Herbaria (CHAH), the APC provides information on a state-by-state basis, but is not synthesised nationally. Recently, the Global Register of Introduced and Invasive Species (GRIIS) for Australia^18^ was published by the Invasive Species Specialist Group (ISSG)^19^ following the methodology proposed by Pagad *et al*.^20^. However, to date, Australia is still lacking a dataset that integrates all the existing data sources across the six Australian states and two main territories (for simplicity hereafter referred to as ‘states’) with those that have been produced at the national scale. In order to deliver standardised up-to-date information and create a unified dataset nationally, an automated system that integrates datasets and specifies plant species origin and introduction status is needed. This will provide a strong evidence-base for planning and informing actions for prevention and to mitigate risks^21^.

In this paper, we collate, combine and unify information from ten independent data sources for all alien plant species recorded in Australia into a unified and standardised national dataset, the Alien Flora of Australia (AFA). By integrating all existing data sources and embedding a technical validation step, this dataset is the first of its kind for Australia.

To construct the AFA, we first had to integrate and standardise terminology on plant invasions across Australia and propose prioritisation systems to tackle mismatches existing at different spatial scales^22^. Then, we had to develop (i) an R script able to combine the information contained within the APC and Australian state plant censuses, and (ii) updated standardised datasets at the jurisdictional level. Afterwards, we integrated all the information into (iii) the Alien Flora of Australia (AFA), a nationally unified dataset. With its associated script, the dataset presented here can be regularly updated with each new release of the state plant censuses. Hence, the Alien Flora of Australia and its associated script enable users to leverage both Australian state and national plant censuses and create customised data subsets suited to their specific needs regarding both area of interest and desired taxonomy. Through its real time coordination and reconciliation of open-access data, the approach used to create the AFA can help transform knowledge, understanding and the efficacy of invasive plant species ecology, management and policy in Australia.

## Methods

**Fig. 1:**
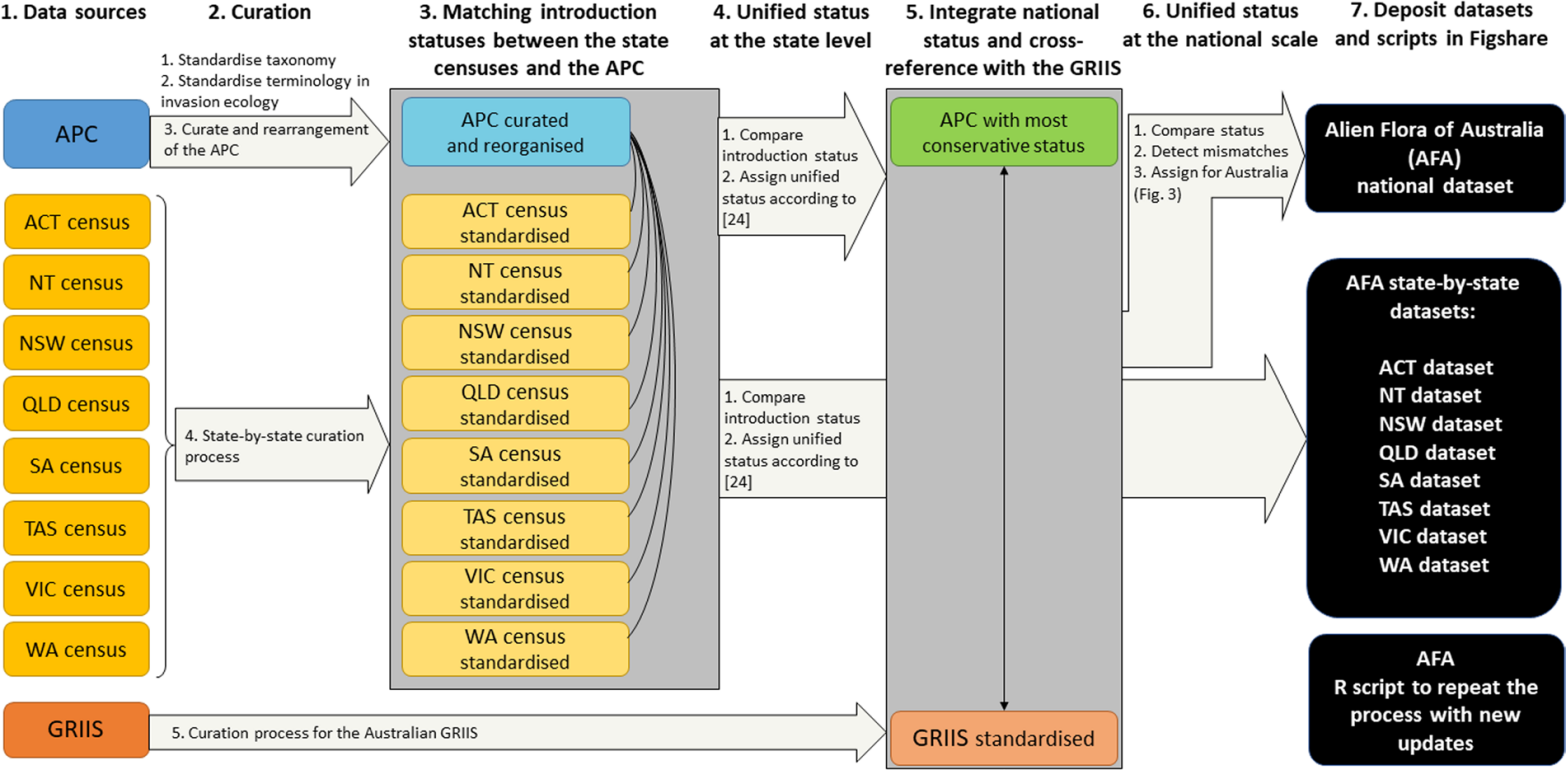
The steps to create the Alien Flora of Australia (AFA) dataset, including obtaining the data sources, curation and standardisation, cross-reference information at the state level and unify introduction statuses at the state level, integrate introduction status information at the national level, cross-reference information at the national level and unify introduction statuses at the national level. APC is the acronym of the Australian Plant Census whereas GRIIS is the acronym of the Global Register of Introduced and Invasive Species. States and main territories have also been abbreviated (the Australian Capital Territory, ACT; New South Wales, NSW; the Northern Territory, NT; Queensland, QLD; South Australia, SA; Tasmania, TAS; Victoria, VIC; Western Australia, WA).

We used R 4.1.1 (R Core Team; https://www.R-project.org/) to create an R script that combines the information recorded on the APC with all the state flora censuses and the GRIIS in order to detect mismatches between species nomenclature and introduction status. The script also allows addressing those mismatches into elucidating a unified introduction status for each species at the national level^22^. The script can be used with future releases of the APC or any of the state floras, to automate this process. There are seven main steps underpinning the creation of the AFA, which we outline here (Fig. 1).

### Data sources (step 1)

The AFA harmonises data from ten independent repositories, which we have combined and integrated using the steps and framework detailed in Fig. 1. Firstly, we obtained the three major types of data sources to be used in the creation of the AFA either via direct download or obtained from authoritative sources. The data sources included: i) the Australian Plant Census (APC); ii) the state flora censuses (eight repositories in total); and iii) the Australian Global Registry of Introduced and Invasive Species (GRIIS) (Fig. 1; Tables 1–3). We only included information on vascular plants; other taxa were excluded at the first step of data curation.

**Table 1:** Sources for state vascular flora censuses, latest version available for each of them, or whether continuously updated, accessed date, and conversion procedure followed in order to standardise the introduction and naturalisation statuses for all states to make them comparable.

State	State code	Comments on the information within each census	Version	Conversion for introduction status standardisation		
				Codes for presence	Codes for Darwin Core establishment means (or equivalent)	Codes for Darwin Core degree of establishment (or equivalent)
Australian Capital Territory	ACT	The census of the Vascular Plants of the ACT includes native and naturalised vascular plants that are known to occur in the ACT but excluding Jervis Bay.	2019	NA	-Codes for ORIGIN: [blank] = native Exotic [Aust] = introduced Exotic [EA] = introduced Indigenous/Exotic [Aust] = native colonising	-Codes for NATURALISED STATUS: Doubtfully = doubtfully naturalised Formerly = formerly naturalised
Citation: Lepschi, B.J., Cargill, D.C., Albrecht, D.E. & Monro A.M. (Eds.) (2019). Census of the Flora of the Australian Capital Territory. Version 4.1 (30 August 2019). Canberra, ACT, Australia. https://www.cpbr.gov.au/cpbr/ACT-census/vascular-gen-alpha.html, accessed August 2022.						
Northern Territory	NT	FloraNT is the main information source for the NT’s flora; it includes information about species origin (native or introduced) and threatened and endemic species	2021	-Codes for Introduced Status: Formerly introduced to NT extinct = formerly introduced	-Codes for Introduced Status: Native to NT = native Introduced to NT = introduced Status uncertain in NT = uncertain origin	NA
Citation: Northern Territory Herbarium (2015). FloraNT Northern Territory Flora Online. Department of Land Resource Management. http://eflora.nt.gov.au/NTSpeciesList?heading=sNTSpecies, accessed August 2022 (version from 2021).						
New South Wales	NSW	The Flora of NSW includes distribution within the state and interstate for plant species recorded in NSW, stating if their origin is natural or introduced.	2022	NA	-Codes for natural/introduced N = native N/I = native colonising I = introduced	NA
Citation: PlantNET (The NSW Plant Information Network System). Royal Botanic Gardens and Domain Trust, Sydney. https://plantnet.rbgsyd.nsw.gov.au, accessed June 2022. The Flora of NSW was obtained directly from the Royal Botanic Gardens. It cannot be directly downloaded from electronic sources. It can be accessed online.						

Please notice that we have specified the codes keeping the original column names for each of the state censuses.

**Table 2:** Sources for state vascular flora censuses, latest version available for each of them, or whether continuously updated, accessed date, and conversion procedure foll and naturalisation statuses for all states to make them comparable (Continuation of Table 1).

State	State code	Comments on the information within each census	Version	Conversion for introduction status standardisation		
				Codes for presence	Codes for Darwin Core establishment means (or equivalent)	Codes for Darwin Core degree of establishment (or equivalent)
Queensland	QLD	The census of the QLD Flora includes name, distribution and status of Queensland native and naturalised plant species.	2022		-Codes for Origin_Status: Native to QLD = native Native and naturalised in QLD = native colonising	-Codes for Origin_Status: Formerly naturalised in QLD = formerly naturalised Doubtfully naturalised in QLD = doubtfully naturalised Naturalised in QLD = naturalised
Citation: Laidlaw, MJ. (2022). Census of the Queensland flora and fungi 2022: Vascular Plants (Print). Queensland Department of Environment and Science, Queensland Government. https://www.data.qld.gov.au/dataset/census-of-the-queensland-flora-and-fungi-2022/resource/548d9394-c94c-4512-a2fa-8f667dac2a2f, accessed May 2023.						
South Australia	SA	The Census of SA Plants, Algae and Fungi includes information on introduction (flagged with *) and threatened categories of plant species in SA	2022	-Code for NPW ACT STATUS COMMENT: When string EX or “extinct” is detected = presumed extinct	-Codes for INTRODUCED: [blank] = native * = introduced	NA
Citation: Department for Environment and Water (2022). Biological Databases of South Australia (BDBSA) Vascular Plant BDBSA Taxonomy. https://data.environment.sa.gov.au/Content/Publications/vascular-plants-bdbsa-taxonomy.xlsx, accessed August 2022.						
Tasmania	TAS	The Census of the Vascular Plants of TAS accounts for every vascular plant taxon, both native (blank entry) and naturalised (flagged with ‘i’), that grows spontaneously in Tasmania	2022	-Code for EXTINCT: x = presumed extinct (if intro is native) or formerly introduced (if intro is naturalised)	-Codes for INTRO: [blank] = native i = naturalised?i = doubtfully naturalised	NA
Citation: de Salas, M.F. & Baker, M.L. (2022) Census of the Vascular Plants of Tasmania, including Macquarie Island. https://flora.tmag.tas.gov.au/resources/census/, accessed November 2022.						

Please notice that we have specified the codes keeping the original column names for each of the state censuses.

**Table 3:** Sources for state vascular flora censuses, latest version available for each of them, or whether continuously updated, accessed date, and conversion procedure followed in order to standardise the introduction and naturalisation statuses for all states to make them comparable (Continuation of Tables 1 and 2).

State	State code	Comments on the information within each census	Version	Conversion for introduction status standardisation		
				Codes for presence	Codes for Darwin Core establishment means (or equivalent)	Codes for Darwin Core degree of establishment (or equivalent)
Victoria	VIC	VicFlora is a comprehensive list of vascular plants of Victoria that is continuously updated; it provides information on origin and introduction status of native and introduced flora based on the Darwin Core terminology	May 2023	-Codes for occurrenceStatus: excluded = [record deleted] extinct = presumed extinct (if establishment_means is native or uncertain) extinct = formerly introduced (if establishment_means is introduced)	-Codes for establishment_means: native = native introduced = introduced uncertain = uncertain origin -For native species, codes for has_introduced_occurrences: 1 = native colonising	-Codes for degree_of_establishment Casual = introduced Established = naturalised Reproducing = naturalised [blank] = uncertain origin (if establishment means is uncertain) [blank] = introduced (if establishment means is introduced)
Citation: VicFlora (2023). Flora of Victoria, Royal Botanic Gardens Victoria. Available online: https://vicflora.rbg.vic.gov.au/flora/download?q=*:*, accessed February 2023.						
Western Australia	WA	FloraBase includes information for native (flagged with N), naturalised (flagged with A) and native colonising (flagged with M) vascular plants in WA	2022	-Codes for IS_CURRENT: N = presumed extinct if it is native or native weed, and formerly introduced if it was introduced (however, the version provided by the WA Herbarium only included present species)	-Codes for NATURALISED_STATUS: N = native M = native colonising	-Codes for NATURALISED_STATUS: A = naturalised
Citation: Florabase was obtained from the Western Australian Herbarium. It cannot be directly downloaded from electronic sources. It can be accessed on: https://florabase.dbca.wa.gov.au/. Florabase—the Western Australian Flora. Department of Biodiversity, Conservation and Attractions. Census obtained in October 2022.						

Please notice that we have specified the codes keeping the original column names for each of the state censuses.

The first and third data sources, the APC and the GRIIS, are at the national scale. The Australian Plant Census (APC) is a list of the Australian flora, both native and introduced; it includes accepted names, synonyms and misapplications for these names, and specifies species distribution on a state-by-state basis for continental Australia and its external territories. Taxa known only from cultivation in Australia are excluded in the APC, and therefore also from the AFA database created here. The Australian GRIIS checklist is a species catalogue for introduced and invasive species recorded from Australia that includes taxonomic information based on the Global Biodiversity Information Facility (GBIF).

The second source of data consisted of the eight plant censuses developed independently by environmental government agencies and herbaria associated with each of the two main Australian territories (Australian Capital Territory (ACT; https://www.cpbr.gov.au/cpbr/ACT-census/vascular-gen-alpha.html) and the Northern Territory (NT; http://eflora.nt.gov.au/NTSpeciesList?heading=sNTSpecies)), and the six Australian states (New South Wales (NSW; https://plantnet.rbgsyd.nsw.gov.au), Queensland (QLD; https://www.data.qld.gov.au/dataset/census-of-the-queensland-flora-and-fungi-2022), South Australia (SA; https://data.environment.sa.gov.au/Content/Publications/vascular-plants-bdbsa-taxonomy.xlsx), Tasmania (TAS; https://flora.tmag.tas.gov.au/resources/census/), Victoria (VIC; https://vicflora.rbg.vic.gov.au) and Western Australia (WA; https://florabase.dbca.wa.gov.au/)) (see Tables 1–3 for details on information included within each state census).

The Alien Flora of Australia (AFA) is available for the latest release of the APC (2022) and the GRIIS v1.9 (2022). Similarly, the state-by-state comparative datasets created here are available for the latest releases of the state plant censuses, ranging from 2019 for the ACT, to the most updated ones for the rest of the states, corresponding to censuses as of 2022 (except for Victoria, where the latest release dated from 2023).

### Curation and standardisation (step 2)

Herbaria from different states follow various versions of protocols for classification, and display the available data inconsistently, therefore limiting data federation. Thus, after obtaining all the data sources we had to standardise them (Fig. 1).

#### Standardised taxonomy

We followed the taxonomy and nomenclature adopted for the APC as the most recognised authority for the Australian vascular flora, which is endorsed by the Council of Heads of Australasian Herbaria (CHAH). In addition, our script allows matching the taxonomic nomenclature used in the APC, to other taxonomies extensively used internationally, such as the GBIF taxonomic backbone (10.15468/39omei) and the standardised nomenclature provided by WorldFlora Online (WFO; http://www.worldfloraonline.org/) and provide all of them in the AFA. We cross-referenced the taxonomy and nomenclature with the one used in the GBIF taxonomic backbone and with WFO, using rgbif^23^ and WorldFlora^24^ R packages, respectively, without fuzzy matching.

#### Standardised terminology in invasion ecology

To address incompatibilities and differences regarding terminology on invasion ecology when cross-referencing information among the data sources, we based the AFA terminology on an adaptation from Blackburn’s framework^8^ described in detail by Martín-Forés *et al*.^22^. Such proposed terminology is the most directly comparable with the one employed on the APC, and it provides information about presence, origin and introduction status in a combined manner. Following the discussion addressed by Martín-Forés *et al*.^22^, native species in the AFA can have one of the following statuses: native, native colonising or native potentially colonising (when they were also naturalised or potentially naturalised, respectively within the same state or territory of origin). Alien species in the AFA can be considered: introduced (to refer to casual aliens or in cases where no information on naturalisation is available), naturalised (when forming self-sustaining populations) – it is stated whether, for the two former ones, the status was doubtfully or formerly when that was the case –, or harmful invasive (when recorded as invasive knowing to cause negative impacts). If the origin for a species is uncertain, it is recorded as ‘uncertain origin’, whereas taxa that are no longer present are recorded as ‘presumed extinct’ (see Tables 4, 5 for definitions).

**Table 4:** Glossary of terms used in the Alien Flora of Australia (AFA) adapted from Blackburn *et al*. (2011).

	Term used in the AFA	Definition at the state level	Definition at the national level
Native (all)	Native	Native to a given Australian state without being naturalised in other areas of such state	Native to at least one Australian state regardless of being introduced or naturalised into other states
	Native potentially colonising	Native to a given Australian state being potentially naturalised in other areas of such state	Native to at least one Australian state in which it is also doubtfully naturalised regardless of being introduced or naturalised into other states
	Native colonising	Native to a given Australian state although being also naturalised in other areas of such state	Native to at least one Australian state in which it is also naturalised regardless of being introduced or naturalised into other states
Alien (all)	Introduced	Species that is an alien and is recorded as introduced into a given state^7,8^	Species that is not native to any Australian state and is introduced in at least one state. There is not specific information of its naturalisation in the combined data sources, therefore it is not possible to know.
	Doubtfully introduced	Species for which it is uncertain if it is introduced in a given state.	Species that is not native to any Australian state and is doubtfully naturalised in at least one state, without being known to be naturalised in any state (there are currently no vascular plant species recorded with this status at national scale).
	Formerly naturalised	Species that was known to have been introduced in the past into a given state. Although it could be presumed to have been eradicated, it would most likely still be a casual alien.	Species that is not native to any Australian state and neither introduced or naturalised nor doubtfully introduced and doubtfully naturalised in any other state. It could be presumed to have been eradicated although it is likely to still be a casual alien (there are currently 40 species under this category at national scale).
	Naturalised	Introduced species that forms unassisted self-sustaining populations^7,8^. The only species that was recoded as ‘reproducing’^12^ in one of the states has been grouped under this category	Species that is not native to any Australian state and is naturalised in at least one state.
	Doubtfully naturalised	Species that despite being introduced, it is unknown if it forms self-sustaining populations. In other sources, sometimes referred to as adventive.	Species that is not native to any Australian state and is doubtfully naturalised in at least one state, without being known to be naturalised in any state.
	Harmful invasive	Invasive alien species (i.e. naturalised species that has dispersed and spread in the introduced range) that has a negative impact within the invaded range and/or to pose a threat to native biodiversity^7^. In the GRIIS referred to as invasive [32–34].	

See full discussion regarding terminology in Martin-Fores *et al*. (2023).

**Table 5:** Glossary of terms used in the Alien Flora of Australia (AFA) adapted from Blackburn *et al*. (2011) (Continuation of Table 4).

	Term used in the AFA	Definition at the state level	Definition at the national level
Other categories	Uncertain origin	Species for which its native area is not known with certainty	Species of unknown origin that occurs in at least one state.
	Presumed extinct	Species that was native to a given Australian state although is now presumed to be extinct	Species that is now presumed to be extinct in at least one Australian state and is not recorded to be present in any other form any other Australian state (there are currently 21 species under this category at national scale).
	Formerly introduced	Species that was known to have been introduced in the past into a given state, but there is no longer present. It could be presumed extinct or have been eradicated.	Species alien to Australia that has now been eradicated or is extinct in at least one Australian state and is not recorded to be present in any other Australian state (there is currently one species under this category at national scale).

See full discussion regarding terminology in Martin-Fores *et al*. (2023).

In addition, the AFA also includes the Darwin Core Standard (https://dwc.tdwg.org/) proposed by the Biodiversity Information Standards (TDWG) to refer to the species origin (*establishment means*; http://rs.tdwg.org/dwc/doc/em/2021-09-01), and the extent to which an introduced species survives, reproduces, and expands in its introduced range (*degree of establishment*; http://rs.tdwg.org/dwc/doc/doe/2021-09-01). By including both the Blackburn’s framework and the Darwin Core standard, we intend for the AFA to be as useful as possible for researchers, biosecurity departments, environmental managers, database managers and herbaria alike.

#### Curation and rearrangement of the Australian Plant Census

We downloaded the APC dataset from the Vascular Plants NSL Service APC^20^ and used the package stringr^25^ to work and manipulate text strings. The information on the column “Distribution” from the APC, included in a consecutive manner for each row all the information about a species presence and origin in all the Australian states and main and external territories in which it was present. We therefore split the information on distribution into individual columns corresponding to each of the Australian States and each of the external territories. The external territories comprised those independent territories in Australia, as well as islands that, despite belonging to one of the state’s jurisdictions, are located far away and therefore have a different flora than the one in the state they are associated with. As such, the external territories included Ashmore Reef (AR), Cartier Island (CaI), Christmas Island (ChI), Cocos Islands (CoI), Coral Sea Islands (CSI), Heard Island (HI), Lord Howe Island (LHI), MacDonald Island (MDI) and Norfolk Island (NI). Although, external territories were excluded from the state-by-state comparison explained below, we still provided, for each of them, the information on the present flora extracted from the APC.

For those species that had a combination of statuses within the same state, we condensed the information into one introduction status per species and state or territory (see Tables 1–3 for details of status conversion). This happened for 0.3% of the vascular plant species records at the national scale. Species recorded as “native and naturalised” or “native and naturalised and uncertain origin” within a certain state were reassigned as ‘native colonising’, as they are native to part of the state, but colonising and therefore considered introduced in other areas within the same state. Similarly, species recorded as “native and doubtfully naturalised” or “native and uncertain origin” were reassigned to ‘native potentially colonising’ (see Martín-Forés *et al*.^22^ for discussion on terminology in invasion ecology across Australia).

#### State-by-state curation process

For the eight repositories at the state scale, we developed standardised datasets (see Martín-Forés *et al*. 2023). To do so, we matched each species canonical name (i.e., species name without the author) displayed in the state plant census with the APC to obtain the equivalent accepted name from the APC for those species that were known synonyms or had been misapplied. We then developed a conversion procedure to standardise all introduction statuses across Australian states (see Tables 1–3 for details). Standardised nomenclature adapted from the unified framework on biological invasions^8^ was used to combine the information about presence (present/extinct), origin (native/introduced/uncertain) and introduction status (casual/ naturalised/ invasive) for all data sources. For example, for a species present, introduced, and for which its degree of establishment according to Darwin Core was known as ‘established’, we recorded it directly as naturalised. To facilitate understanding of the different terminology when used at both state and national level, we have provided a glossary with specific meanings for each term at both scales and according to different sources of vocabulary for invasion ecology (Tables 4, 5). Details for rationale behind this decision and discussion on equivalences between different frameworks and standards can be found in Martín-Forés *et al*.^22^.

#### Curation process for the Australian GRIIS

The Australian GRIIS is structured in three different datasets that were downloaded from (https://cloud.gbif.org/griis/resource?r=griis-australia; GRIIS v1.9, accessed February 2023). The three datasets were merged into one and only the records for plants were kept. The taxa that appeared recorded as ‘invasive’ based on impact were changed to ‘harmful invasive’ according to the definitions provided in detail by Essl *et al*.^26^ and Martín-Forés *et al*.^22^.

### Matching introduction statuses between the state censuses and the APC (step 3)

In each of the datasets at the state level, we matched the corresponding accepted names in APC to the complete APC dataset to obtain the corresponding state introduction status in the APC (Fig. 1).

We also matched the taxon status to identify the species that have been excluded from the APC, and those that were recorded in the APC as pro-parte or pro-parte misapplied, so that we could treat these cases individually. Pro-parte or pro-parte misapplied species are, respectively, those that can be both an accepted name and synonym for another accepted name, or that correspond to two different accepted names, and those that have been mis-assigned to different accepted species. If pro-parte and pro-parte misapplied species only matched one accepted species from the APC, we kept those records. However, in cases where they matched two or more accepted species from the APC, we stated that it was impossible to elucidate to which one the species name appearing on the state census would correspond.

Once matched, the distribution and introduction status within each Australian state is scored by cross-checking extracted information from the APC and the state flora censuses accessed from the state herbaria. As such, we compared species scientific name (including authorship), canonical name, and introduction status recorded on the state census with those recorded on the APC (See supplemental Table S1 in Supplementary material for details). This comparison between the state plant censuses and the distribution information recorded on the APC detected potential mismatches (see Martín-Forés *et al*.^22^ for discussion on mismatches in introduction status at the state and territory levels in Australia).

### Assigning a unified status for a given species in each state (step 4)

The script we created easily identified and tackled mismatches in nomenclature, introduction status or even presence within a certain area (Fig. 1). Where introduction statuses differed between APC and the state plant census, we applied the prioritisation procedure proposed in the integration exercise conducted by Martín-Forés *et al*.^22^ to address inconsistencies regarding introduction status at the jurisdictional level in Australia. According to such prioritisation procedure, the introduction status that has advanced the furthest along the continuum prevails. This was decided as a precautionary measure for addressing potential invasion and to be as conservative as possible when designing management and eradication programs. As such, when a species was not listed on the APC or was recorded on the APC as not present in a given state, we kept the introduction status recorded in the state plant census. For species that appeared in both sources but displayed a mismatch in the introduction status, we prioritise the most conservative introduction status (i.e. the one advanced the furthest along the continuum^22^). In all component datasets developed at the state level, we incorporated a new column with the unified status for each species in such state (See Figshare to access all the standardised state-by-state datasets for all Australian jurisdictions^27^).

### Integrating unified state statuses into a national status and cross-reference with the GRIIS (step 5)

The unified status for each species across all Australian states were combined and integrated into a status at the national level (Fig. 1). For each accepted species on the APC dataset, we matched the unified status obtained for each state in each of the standardised datasets at the jurisdictional level. When the species did not appear listed on the state census, we kept the introduction status recorded on the APC; when the species was not originally listed on the APC, but only on the state census, we did not incorporate it at national level, therefore appearing only on the specific standardised datasets for the corresponding jurisdiction (Figshare^27^). Finally, when the species was recorded in both sources, we integrated the introduction statuses among states into an introduction status at the national scale. To conduct such integration at the national scale, we followed the prioritisation procedure described by Martín-Forés *et al*.^22^ to address inconsistencies regarding introduction status at the national level. The prioritisation procedure at the national scale differed from the one used at the state level as follows: if a species was native to at least one state, it was considered native at the national scale. If it was not ‘native’ to any state, but it was recorded as ‘native colonising’ (or native potentially colonising) in any state, it was considered as native colonising at the national scale. If a species was neither native in any possible form to any state, nor having uncertain origin, then the introduction status that had advanced the furthest along the continuum was the one prevailing at the national scale. Only if the species was not present in any state was it then recorded as presumed extinct at the national scale (see Martín-Forés *et al*.^22^ for detailed prioritisation procedure at the national scale).

We then cross-referenced the unified introduction status we obtained at the national scale with the one appearing on the Australian GRIIS^21^ and harmonised the information recorded in both into the AFA status at national scale (See Supplementary material for details).

### Assigning a unified status (AFA status) for a given species at the national scale (step 6)

Where introduction status differed between the national status and the GRIIS, we followed the steps proposed by Martín-Forés *et al*.^22^ (Fig. 1).

For species for which the introduction status resulting from combining state data sources at the national scale was alien in any form, and for which the status recorded on the GRIIS was ‘invasive’, the unified AFA status was stated as ‘harmful invasive’. This was due to the fact that the GRIIS classifies invasive species based on negative impact, not only on distribution along the continuum (see Martín-Forés *et al*.^22^ for detailed discussion). When other mismatches were identified (e.g., species that are native to at least one Australian state but appeared recorded as introduced or invasive (i.e., harmful invasive) in the GRIIS), the AFA status corresponded to the one resulting from integrating the state statuses at the national scale as detailed in the previous step.

### Depositing the R script, standardised state-by-state datasets and the AFA dataset in open repositories (step 7)

Finally, we made sure the R script was accessible both via Figshare and github to facilitate updated versions of the AFA with new releases of the state censuses or the APC (Fig. 1).

The standardised datasets at the state level are accessible for informative purposes or in cases where jurisdictional authorities can make use of the information provided there.

The AFA dataset contains a comprehensive up-to-date information on alien flora at the national level.

## Data Records

The dataset is available at Figshare, 10.6084/m9.figshare.23513478^27^. The data records are structured as follows:

The AFA national dataset is available for direct download in a.csv file. The AFA national dataset contains information on taxa canonical and scientific names, authorship, taxonomic rank (e.g. species epithet, subspecies epithet), and the taxon status according to the APC (which in all cases is accepted for this dataset), the unique ID status for each taxon is also provided. Moreover, there are columns with the unified introduction status for all taxa in each state and main territory, and with the introduction statuses populated from the APC for the external territories. Afterwards, there is the national introduction status resulting from the integration at the national scale, the GRIIS status and a column resulting from the comparison between the two. Finally, there is the AFA unified status that corresponds to the unified status for each taxon across all data sources at the national scale. In addition, the AFA unified status has been converted into its corresponding Darwin Core equivalences for the terms establishment means and degree of establishment.

We provide four more folders in Figshare^27^. The first folder is a zip folder called AFA_state-by-state datasets that contains all the state-by-state standardised datasets (eight in total) in.csv format which were produced as intermediate steps for creating the AFA national dataset. The state-by-state standardised datasets are all displayed following the same structure, including information on the taxonomy recognised by the state censuses and the APC, the introduction status recorded on both the state census and the APC, the taxon status according to APC (e.g. accepted, misapplied, excluded), a column resulting from the comparison of introduction status between the state census and the APC, and a column with the unified introduction status at the state level. In addition, the unified introduction status at the state level was converted into its corresponding equivalences from the Darwin Core, establishment means and degree of establishment terms.

The second zip folder is called AFA_metadata and contains two.csv with the metadata for the AFA national dataset and the state-by-state datasets.

The third folder is a zip folder called AFA_R_Scripts that contains three R files which were produced as intermediate steps for creating the AFA national dataset. The AFA_main_R_script contains the condensed information on how to reproduce the process to create the AFA dataset. In such instructions, we duplicated the first step to allow for users to either replicate the static copy or the AFA presented here, or alternatively to create updated versions in the future with new releases of the APC and the state censuses. In addition, there are two more R files containing the primary and secondary functions to be used in the main script. These two files containing the functions can be easily loaded following the steps detailed on the main R script. We split the files, so that in the future, the script developed to create this dataset can be easily adapted as needed and reused to create updated versions of the AFA with new releases of the state and main territory plant censuses, and of the Australian Plant Census. The three R files are also accessible via github (https://github.com/MartinFores/AFA).

Finally, the fourth zip folder is called AFA_raw_data and it contains the raw versions obtained from the different data sources that were employed to create the AFA national dataset presented here, together with a pdf file outlining the corresponding citations for each data source. As such, the users can reproduce the AFA national dataset presented here, by accessing a static copy of the APC, the three datasets conforming the GRIIS, and static copies of the state and main territory plant censuses for the corresponding versions detailed on Tables 1–3.

## Technical Validation

This standardised system is itself the result of a technical validation. By cross-referencing all up-to-date official sources of vascular plant censuses in each of the Australian states, main territories and at the national scale, we have identified all the existing mismatches to date, and when a mismatch has been detected, the tool ensures that the most conservative approach is followed.

When the metadata of certain state census appeared unclear, we contacted the relevant authority to ensure accuracy in the interpretation of the terminology employed and subsequently in our conversion.

Additionally, we consulted with a subcommittee of the Council of Heads of Australasian Herbaria (CHAH), the Herbarium Information Systems Committee (HISCOM; https://chah.gov.au/the-herbarium-information-systems-committee/), and each of the data custodians for each state. We took this step to ensure the conversion of introduction statuses was accurate. As a result of this process, we obtained feedback to incorporate the Darwin Core terminology in our standardised system to also be inclusive of other international standards that may be implemented in the foreseeable future.

### Supplementary information

Supplementary material for

## Data Availability

Our code is fully available in Martín-Forés *et al*. (2023); 10.6084/m9.figshare.23513478^27^. The code can also be accessed on github, https://github.com/MartinFores/AFA.
