# Supplementary material for: The Alien Flora of Australia (AFA), a unified Australian national dataset on plant invasion
Source: Sci Data. 2023 Nov 27;10:834. doi: 10.1038/s41597-023-02746-3 (PMC10682373; doi:10.1038/s41597-023-02746-3)
Supplement: Supplementary file 1 — Supplementary material for [file 41597_2023_2746_MOESM1_ESM.docx]

**Supplementary material**

**Supplemental Table S.1. Metadata with explanations for comparison columns at the jurisdictional level (i.e. between the state flora censuses and the Australian Plant Census (APC)), and at the national level (i.e. between the APC and the Global Register of Introduced and Invasive Species (GRIIS)).**

|  |  | **Comparison comment** | **Meaning** |
| --- | --- | --- | --- |
| Comparison at the jurisdictional level | State plant censuses vs. APC | Similar for the state flora and the APC | Species for which its introduction status was the same in both datasets. |
|  |  | Status not assigned on the state flora | Species that, despite being recorded on the state census, it did not have an introduction status assigned |
|  |  | Not listed on the APC with the name appearing on the state flora | Species that did not appear at all on the APC therefore with no matches |
|  |  | Not recorded on the APC as present in this state | Species for which we found a matched record on the APC but such state or main territory did not appear in the distribution column |
|  |  | According to the state flora is [corresponding introduction status], while according to the APC is [corresponding introduction status] | Species that is recorded on the APC and present in such state but there is a mismatch in the introduction status (either in establishment means or degree of establishment according to the Darwin Core) |
|  |  | Taxon excluded on the APC | Species that appeared as excluded taxa on the APC |
|  |  | It corresponds to several accepted names on the APC due to have been pro parte misapplied, therefore it is impossible to assign one | Pro-parte and pro-parte misapplied species that matched several accepted species on the APC |
| Comparison at the national level | National status vs. GRIIS | Similar for our national status and the GRIIS | Species for which its introduction status is the same for our national status and the GRIIS |
|  |  | Not listed on the GRIIS with the name appearing on the APC or not recorded as introduced at the national scale | Species that did not appear with such name on the GRIIS |
|  |  | Not recorded in Australian states, only in external territories | The species appears on the GRIIS but according to the APC is only present in external territories, not in states and main territories |
|  |  | According to our national status is [corresponding introduction status], while according to the GRIIS is [corresponding introduction status] | The species is recorded on the APC and the GRIIS but there is a mismatch in the introduction status obtained via our unified system (either in establishment means or degree of establishment according to the Darwin Core). |
